# Supplementary material for: The Vulvar Cancer Risk in Differentiated Vulvar Intraepithelial Neoplasia: A Systematic Review
Source: Cancers (Basel). 2021 Dec 7;13(24):6170. doi: 10.3390/cancers13246170 (PMC8699429; doi:10.3390/cancers13246170)
Supplement: Supplementary file 1 [file cancers-13-06170-s001.zip › cancers-1456277-supplementary.pdf]

cancers

MDPI

Supplementary Materials: The Vulvar Cancer Risk in Differentiated Vulvar Intraepithelial Neoplasia: A Systematic Review

Féline O. Voss, Nikki B. Thuijs, Ravi F. M. Vermeulen, Erica A. Wilthagen, Marc van Beurden and Maaike C. G. Bleeker

Table S1. Search strategy for each database.

Database	Search strategy
Medline	(differentiated or HPV-independent).ti,ab,kf. and ((exp vulvar Neoplasms/ and exp Carcinoma in Situ/) or ("vulvar intraepithelial" or "vulval intraepithelial" or VIN).ti,ab,kf.) or (dVIN or d-VIN).ti,ab,kf.
Embase (Ovid)	(differentiated or HPV-independent).ti,ab,kw. and ((exp vulva tumor/ and exp carcinoma in situ/) or ("vulvar intraepithelial" or "vulval intraepithelial" or VIN).ti,ab,kw.) or (dVIN or d-VIN).ti,ab,kw.
Scopus	TITLE-ABS-KEY(((differentiated or HPV-independent) and ("vulvar intraepithelial" or "vulval intraepithelial" or VIN)) or dVIN or d-VIN)
